# Thermal Degradation and Flame Retardant Mechanism of the Rigid Polyurethane Foam Including Functionalized Graphene Oxide

**DOI:** 10.3390/polym11010078

**Published:** 2019-01-06

**Authors:** Xuexi Chen, Junfei Li, Ming Gao

**Affiliations:** 1 School of Safety Engineering, North China Institute of Science and Technology, Box 206, Yanjiao, Beijing 101601, China; xuexichen1210@163.com (X.C.); junfeili0304@163.com (J.L.); 2 School of Environmental Engineering, North China Institute of Science and Technology, Box 206, Yanjiao, Beijing 101601, China

**Keywords:** graphene oxide, rigid polyurethane foam, thermogravimetric analysis, activation energies

## Abstract

A flame retardant rigid polyurethane foam (RPUF) system containing functionalized graphene oxide (fGO), expandable graphite (EG), and dimethyl methyl phosphonate (DMMP) was prepared and investigated. The results show that the limiting oxygen index (LOI) of the flame-retardant-polyurethane-fGO (FRPU/fGO) composites reached 28.1% and UL-94 V-0 rating by adding only 0.25 g fGO. The thermal degradation of FRPU samples was studied using thermogravimetric analysis (TG) and the Fourier transform infrared (FT-IR) analysis. The activation energies (*E_a_*) for the main stage of thermal degradation were obtained using the Kissinger equation. It was found that the fGO can considerably increase the thermal stability and decrease the flammability of RPUF. Additionally, the *E_a_* of FRPU/fGO reached 191 kJ·mol^−1^, which was 61 kJ·mol^−1^ higher than that of the pure RPUF (130 kJ·mol^−1^). Moreover, scanning electron microscopy (SEM) results showed that fGO strengthened the compactness and the strength of the “vermicular” intumescent char layer improved the insulation capability of the char layer to gas and heat.

## 1. Introduction

Rigid polyurethane foam (RPUF) is a porous material, and has good shock absorption, low water absorption, low thermal conductivity, and high compressive strength [1,2,3,4]. In recent years, RPUF has been widely used as a structural and insulation material [5,6]. However, compared with inorganic materials, RPUF has low density, a large surface area and easy combustion [7,8,9]. Therefore, it is important to improve RPUF’s flame retardation performance to increase its popularity. Many scholars have done a lot of experimental studies, which aim to improve the fire behavior and thermal stability of RPUF. Compounds containing halogens are good flame retardants, such as tris(2-chloropropyl) phosphate (TCPP) and decabromodiphenyl ethane (DBDPE) [10,11]. However, halogen-containing RPUF will release excessive amounts of toxic gases and smoke during burning, which will seriously endanger human health [12]. Therefore, it is necessary to find an alternative to halogen flame retardants. Expandable graphite (EG) is also a novel intumescent flame retardant. It is not only a very good flame retardant, but also has positive characteristics, such as being low-cost and environmentally friendly. Research has shown that EG played an important role in the condensation phase mainly through the formation of expansive char layer at high temperature [13,14]. However, EG is added to RPUF, which makes the foam loose and polycellular, and deteriorates the mechanical properties of RPUF. Therefore, many researchers have focused on studying the synergistic effects of EG and phosphate, which have shown good results of flame retardation using polyurethane [15,16,17]. 

Graphene is a two-dimensional material consisting of carbon atom layers arranged in honeycomb networks and show impressive mechanical, thermal, optical, and electron transport properties [18,19,20,21]. It is considered to be a promising multifunctional nano-filler polymer. In recent years, many scientists believed that graphene and its derivatives were potential flame retardants with good flame retardation performance. In particular, they along with conventional flame retardant fillers are a promising way to apply to flame retardant polyurethane [22,23,24,25,26,27]. Bao et al. made use of in situ polymerization to functionalize graphene oxide, and applied it to the PS matrices, which dramatically decreased the peak of heat release rate (PHRR), total heat release (THR), peak CO_2_ release rate, and peak CO release compared to those of pure PS [23]. Gavgani et al. reported that graphene oxide (GO), working synergistically with the intumescent flame retardant (IFR) polyurethane, improved the burning behavior of composites [24], and the results showed that employing 2 wt % GO along with 18 wt % IFR (IFR/RPUF composite) obtained the limiting oxygen index (LOI) value of 34.0 and UL-94 V-0 rating. Chen et al. prepared RPUF composites with 14.75 wt % MPP and 0.25 wt % GO, which presented good flame retardancy, and the results of cone calorimeter tests (CONE) showed decreased PHRR, THR, and total smoke production (TSP) compared to those of the pure RPUF [26].

It is very effective to mix trace amount of GO with different flame retardants for obtaining RPUF with low flammability. In a previous work, FRPU/fGO composite was successfully prepared and the results showed that the flame-retardancy and mechanical properties of the composite dramatically improved. Meanwhile, its LOI value reached 28.1% by adding only 0.25 phr fGO and 10 phr EG/DMMP. The tensile strength, elongation at break, and compressive strength of FRPU/fGO composite increased by 41.0%, 50.6%, and 30.0%, respectively [27]. In this study, the effects of graphene oxide and functionalized graphene oxide on the thermal properties and flame retardation mechanism of the flame-retardant-polyurethane systems (FRPU) were investigated using thermogravimetric analysis (TG), Fourier transform infrared spectroscopy (FT-IR), and scanning electron microscopy (SEM).

## 2. Experimental 

### 2.1. Materials

Polyether polyol (including polyols, blowing agents, surfactant and other modifiers, Cst-1076-B) and isocyanates (Cst-1076-A) were purchased from Shenzhen Keshengda Trading Co., Ltd., Shenzhen, China. Sulphuric acid (H_2_SO_4_, 98% AR), hydrogen peroxide (H_2_O_2_, 30% AR), potassium permanganate (KMnO_4_, 99% AR), and boric acid (H_3_BO_3_, 99% AR) were purchased from Tianjin Fuchen Chemical Reagent Co., Ltd., Tianjin, China. DMMP was purchased from Tangshan Yongfa flame retardant materials factory (Tangshan, China). Graphite powder (98.0%, C) was obtained from Tianjin Zhiyuan Chemical Reagent Co., Ltd., Tianjin, China. EG (ADT150, 92%) was purchased from Shijiazhuang Ke Peng flame retardant material factory (China). Furthermore, 3-aminopropyltriethoxysilane ((C_2_H_5_O)_3_–Si–(CH_2_)_3_NH_2_, 98% GR) was supplied by Guangzhou Zhongjie Chemical Technology Co., Ltd., Guangzhou, China.

### 2.2. Sample Preparation

GO was prepared from graphite powder using the Hummers method [28], whereas fGO was prepared using the method reported in a previous work [27]. The GO (1.25 g) was dispersed in 50 mL of ethanol aqueous solution and stirred for 60 min using ultrasonic agitation treatment at 25 °C. Then, 1 mL of 3-aminopropyltriethoxysilane was added to the GO solution, and the mixed solution was stirred well for 0.5 h at 25 °C. Additionally, H_3_BO_3_ (0.5 g) was added to the mixed solution with continuous stirring for 60 min at 25 °C. Finally, the mixture was washed 3 times with ethyl alcohol using suction filtration, and the residuum dried at 60 °C for 24 h. Finally, the RPUF samples were prepared with different formulations (see Table 1) [27].

### 2.3. Testing

The FT-IR spectra of the specimens were obtained on an FTS 2000 FT-IR (Varian, Ok, USA) operated at 1 cm^−1^ resolution within the wavelength range of 4000–400 cm^−1^. The specimen size for the LOI measurement was 130 × 10 × 10 mm^3^ using JF–3 LOI apparatus (Nanjing Jiangning Analytical Instrument Factory, City, China) according to the ASTM D 2863-97. The LOI measurements for each specimen were repeated three times. The data were reproducible within ±1%. Thermogravimetric analysis of the RPUF specimens was performed using a HCT2 thermal analyzer under air and nitrogen atmosphere at a heating rate of 10 °C·min^−1^. All the tests were repeated three times. During the test, 5.0 mg of sample was put in an alumina crucible and heated from ambient temperature to 700 °C. The heating rates were successively varied through values of 5, 10, 15, and 20 °C·min^−1^ under a nitrogen flow rate of 30 mL·min^−1^. The morphologies of the residues obtained from the cone calorimeter test were studied using scanning electron microscopy (SEM, KYKYEM-3200, KYKY, Beijing, China).

## 3. Results and Discussion

### 3.1. Flame Retardancy of RPUF Specimens

Flame retardancy of RPUF is generally evaluated using LOI, vertical burning test (UL-94), and CONE. Their results were reported in a previous work. Its LOI value reached 28.1% after the addition of 10 phr EG/DMMP and 0.25 phr fGO. The UL-94 test reached V-0 rating. In addition, the results of CONE showed that the heat release and the harmful and toxic gas release decreased. The PHRR and THR decreased from 272 to 182 kW/m^2^ and from 47 to 35 MJ/m^2^, respectively. Furthermore, the TSP also dropped from 11.5 to 8.5 m^2^/ m^2^ [27]. 

### 3.2. Thermal Stability of RPUF Specimens 

**Figure 1:**
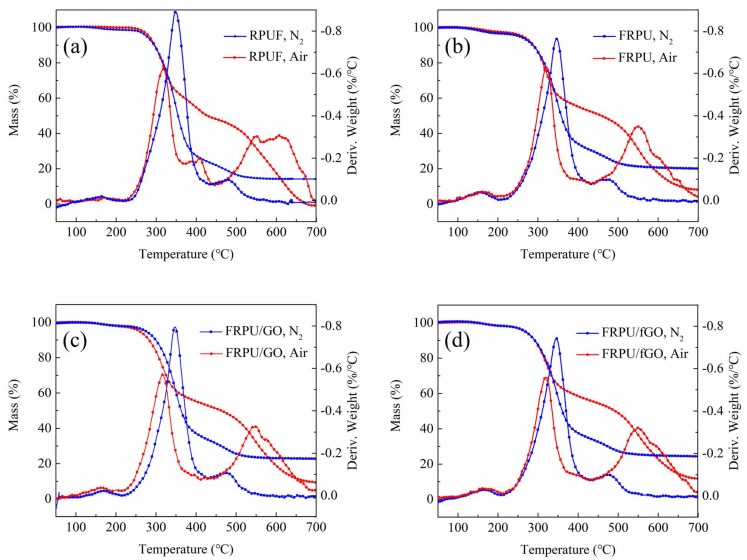
TG and DTG curves of the specimens under nitrogen and air atmospheres: (**a**) rigid polyurethane foam (RPUF); (**b**) flame-retardant-polyurethane systems (FRPU); (**c**) FRPU/ graphene oxide (GO); and (**d**) FRPU/ functionalized graphene oxide (fGO).

In order to investigate the thermal stability of RPUF specimens, TG analysis of the foams was carried out. The TG curves of RPUF specimens under nitrogen and air atmospheres were obtained and are shown in Figure 1. The corresponding TG data is listed in Table 2. The temperature of 5.0% degradation was defined as the initial decomposition temperature (*T_ini_*), and the temperature at which the degradation rate reached its maximum value was regarded as *T_max_*.

As shown in Figure 1 and Table 2, the thermal degradation of RPUF specimens in air atmosphere can be divided into three steps. In pure RPUF curves, during the range of 110–140 °C, some mass loss occurred due to the volatilization of water vapor in the specimen. The temperature range of the second degradation step was within the range of 240–450 °C, which is mainly attributed to monomer precursors, such as polyurethane polyols and isocyanates. Subsequently, the isocyanate dimerizes to form carbodiimide, accompanied by the evolution of volatile compounds, such as CO_2_, CO, alcohols, amines, and aldehydes. The temperature range of the third degradation step was 450–700 °C, which is mainly due to the degradation of substituted urea that is formed due to the reaction of carbodiimide with alcohol or water vapors [2]. 

Compared with the pure RPUF, the thermal degradation of rest of the specimens is similarly processed. However, in the flame retardant systems, their maximum degradation temperature for the first degradation stage reached the value of more than 160 °C, which was about 30 °C higher than that of the pure RPUF. In addition, the results presented in Table 2 showed that the *T_ini_* values of the RPUF specimens were found in the following ascending order: FRPU < FRPU/GO < FRPU/fGO < RPUF. In the second and third steps of flame retardant systems, the *T_max_* values were approximately 320 °C and 550 °C, respectively, which were higher than those of the pure RPUF. This is due to the interaction of EG, DMMP, and nanomaterials in FRPU systems. 

However, the results presented in Figure 1 and Table 2 showed that the thermal degradation of RPUF specimens under a nitrogen atmosphere was mainly within the range of 220–430 °C. In a nitrogen atmosphere, *T_ini_* of the FRPU specimen reduced from 276 and 256 °C, which is due to the addition of DMMP. When GO or fGO was added to the FRPU, the *T_ini_* value increased by 13 °C and 22 °C, respectively. In addition, the *T_max_* values of the RPUF specimens were similar to each other.

As far as the residue yield was concerned, the residue yield of the pure RPUF was less than 0.5% at 700 °C, and the residue yields of other specimens (FRPU, FRPU/GO, and FRPU/fGO) were 7.0%, 9.5%, and 12.5% in air, respectively. It is clear that the residue yields of RPUF specimens were in the following ascending order: RPUF < FRPU < FRPU/GO < FRPU/fGO. A similar rule for the residue yield was observed in a nitrogen atmosphere. 

According to the above description, it was shown that the thermal stability of flame retardant systems, especially of fGO, significantly increased due to nanomaterials in both the air and nitrogen atmospheres during their thermal degradations. 

### 3.3. Decomposition Activity Energies

**Figure 2:**
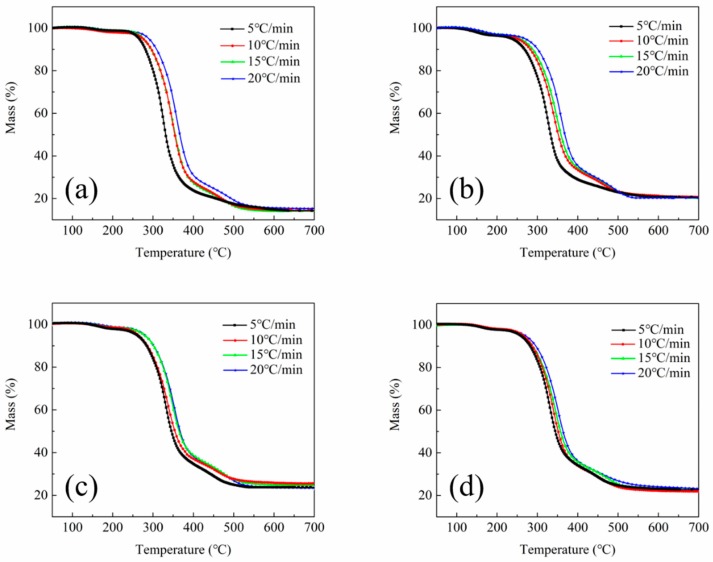
TG curves of the RPUF specimens in a nitrogen atmosphere at the heating rates of 5, 10, 15, and 20 °C·min^−1^: (**a**) RPUF; (**b**) FRPU; (**c**) FRPU/GO; and (**d**) FRPU/fGO.

In order to obtain a better understanding of the degradation process and the effects of GO and fGO on the thermal stability of RPUF, the decomposition activity energies of RPUF specimens were calculated using the equation of Kissinger [29]. The TG curves of RPUF specimens in a nitrogen atmosphere at the heating rates of 5, 10, 15, and 20 °C·min^−1^ are shown in Figure 2. According to the Kissinger’s method, the activation energies (*E_a_*), temperature of the maximum reaction rate at a constant heating rate (*T_m_*), and heating rate (Φ) are correlated using Equation (1).

(1) $$\frac{d\ln\left(\Phi /T_{m}^{2}\right)}{d\left(1/T_{m}\right)}=\frac{-E_{a}}{R}$$

**Figure 3:**
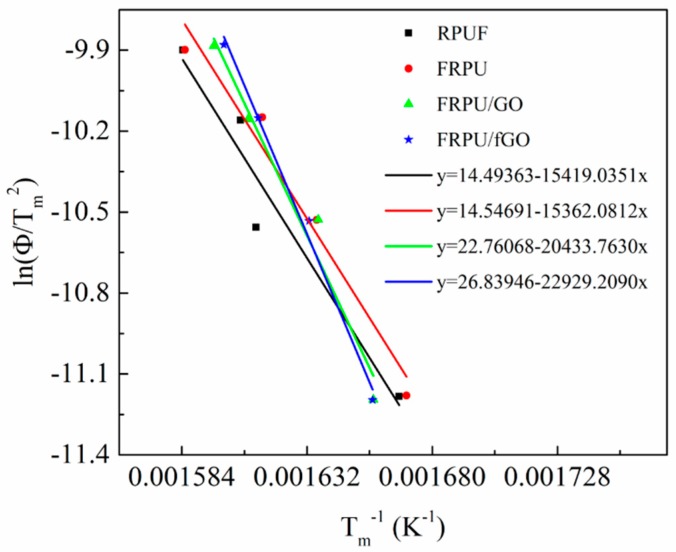
Kissinger method applied to the experimental TG data of RPUF specimens at different heating rates under a nitrogen atmosphere.

From the slope of the plot of ln(Φ/*T_m_*^2^) versus 1/*T_m_*, *E_a_* can be calculated (*E* = *R* × slope). The calculation process is shown in Figure 3. Table 3 presents the activation energies (*E_a_*) of various RPUF specimens. As observed from the results presented in Table 3, the *E_a_* for the decomposition of RPUF is 130 kJ·mol^−1^, while that of EG/DMMP/RPUF (FRPU) is 128 kJ mol^−1^, which shows a drop of around 2.0 kJ·mol^−1^ and may be due to the catalytic effect of EG/DMMP on the decomposition and carbonization of RPUF. Additionally, the *E_a_* values of FRPU/GO and FRPU/fGO are much higher than those of FRPU, and have the values of 167 kJ·mol^−1^ and 191 kJ·mol^−1^, respectively. Generally, the higher the activation energy, the more difficult the degradation of the material. The thermal stability of fGO is better than that of GO, which shows that fGO has better efficiency to increase the thermal stability of RPUF.

### 3.4. FT-IR Analysis of the Residues Heated to Specific Temperatures

**Figure 4:**
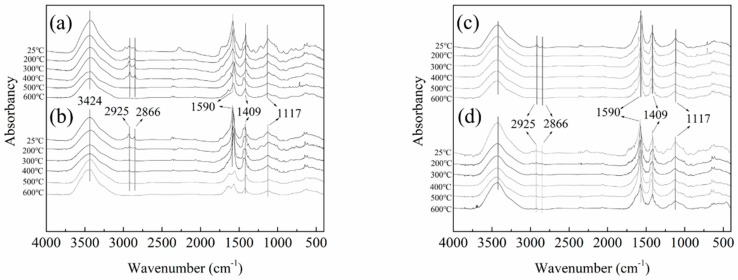
Fourier transform infrared (FT-IR) spectra of the RPUF specimens obtained at specific temperatures: (**a**) RPUF; (**b**) FRPU; (**c**) FRPU/GO; and (**d**) FRPU/fGO.

In order to further study the process of thermal degradation of RPUF specimens, the residues of RPUF, FRPU, FRPU/GO, and FRPU/fGO were obtained by heating the specimens to specific temperatures under a nitrogen atmosphere. The specific temperatures were set to be 200, 300, 400, 500, and 600 °C. The FT-IR spectra of the residues are presented in Figure 4. 

As can be seen from Figure 4, the FT-IR spectra of all samples show a similar absorption peak at 25 °C. The 2925 cm^−^^1^ and 2866 cm^−1^ absorption peaks correspond to stretching mode of C–H in CH_2_ and CH_3_, respectively [30]. The peaks around 1590 cm^−1^ are assigned to the vibration of the aromatic ring [31]. The peaks around 1409 cm^−1^ are typical for the deformation vibration of CH_2_. Additionally, the peak near 1117 cm^−1^ belongs to the C–O–C stretching vibration [32]. With the increase in temperature, the changes in FT-IR spectra for the four samples are found to be similar. However, when the temperature increases to 500 °C, there are nearly no absorption peaks near 2925 cm^−1^ and 2866 cm^−1^ in the FT-IR spectra of pure RPUF and FRPU. Furthermore, the weakened intensities of the bands at 1590 cm^−1^ and 1409 cm^−1^ are caused by the gradual degradation of the molecular chain. As for FRPU/GO and FRPU/fGO, these peaks still exist and are hardly weakened. Furthermore, the stretching vibration of C–H in methyl and methylene of FRPU/fGO specimen is well preserved at 600 °C, which indicates that the addition of fGO increases the thermal stability of FRPU at high temperature. This result is consistent with the conclusions obtained from the thermogravimetric analysis. Therefore, the higher heat resistance of FRPU/fGO specimen indicates that there is a synergistic effect between fGO and EG/DMMP. 

### 3.5. Digital Photos and SEM Images of Char Residue

**Figure 5:**
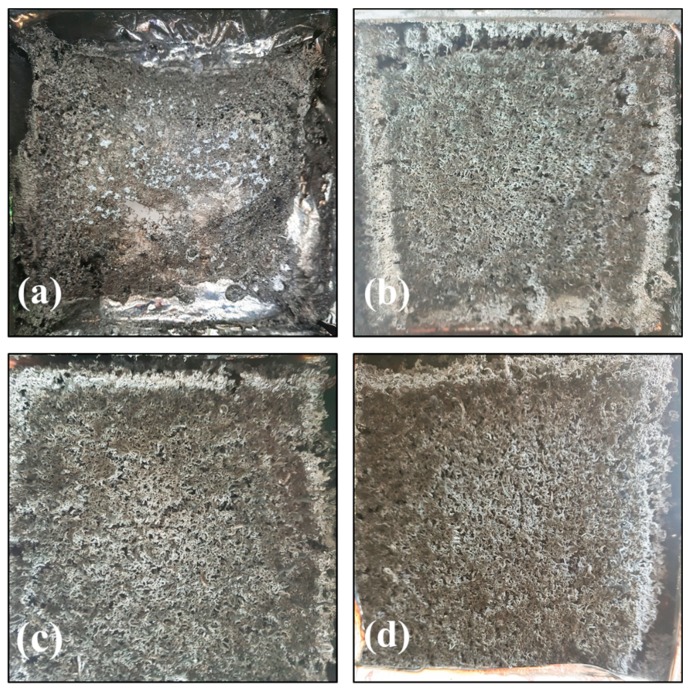
Digital photos of the residues of RPUF samples: (**a**) RPUF; (**b**) FRPU; (**c**) FRPU/GO; and (**d**) FRPU/fGO.

**Figure 6:**
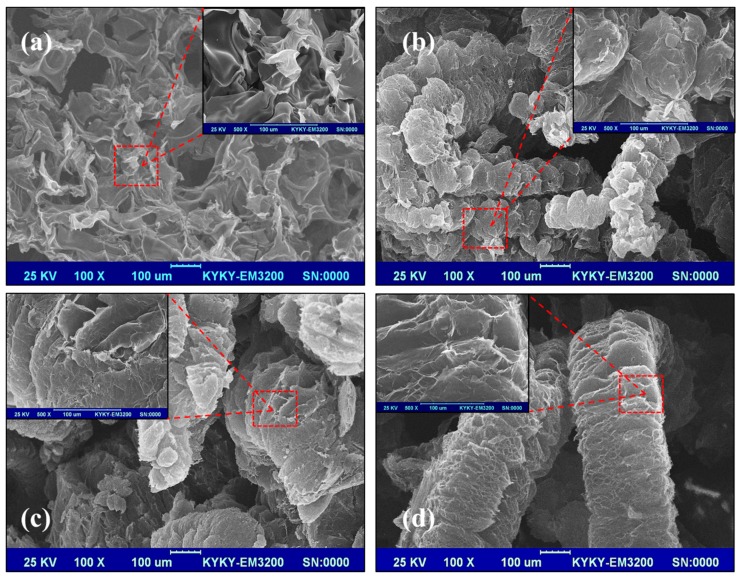
Scanning electron microscopy (SEM) images of the residues of RPUF samples: (**a**) RPUF; (**b**) FRPU; (**c**) FRPU/GO; and (**d**) FRPU/fGO.

In order to elucidate the possible flame retardant mechanism of the condensed phase, the burnt samples were carefully observed. Figure 5 and Figure 6 show the digital photos and SEM images of the residues of RPUF, FRPU, FRPU/GO, and FRPU/fGO samples collected after the CONE, respectively. 

From the macroscopic digital photos (Figure 5), the degree of carbonization of char residues obviously improved with the addition of flame retardant and nano-filler. It can be observed that the macroscopic surface morphology mainly exists in the “vermicular” expanded carbon layer, except for the pure sample. This is because the flame retardants of FRPU samples mainly consist of EG, which expands in volume under high temperature to form “vermicular” expanded carbon layer [33]. When EG and DMMP are compounded, the compactness and strength of the “vermicular” expanded carbon layer will increase, which is due to the reason that DMMP produces phosphoric acid and polyisophosphoric acid of a nonvolatile viscous liquid membrane at high temperature. At the same time, for the micromorphology of char-formed FRPU samples (as shown in Figure 6), the addition of DMMP makes the char layer thicker and denser after combustion, and there are basically no holes on the surface of the carbon layer. Especially, the “vermicular” surface of FRPU/GO and FRPU/fGO obviously became flatter and more continuous compared to the FRPU sample. Furthermore, the “vermicular” expanded char layer becomes dense and complete, resulting in more unbroken vesicles on the underlying surface of the char layer. The results showed that the nanomaterials enhanced the compactness and strength of “vermicular” expanded char layer, improved the insulation ability of the char layer to gas and heat, and significantly improved the flame retardant property and smoke suppression effect of the foam. Particularly, the FRPU sample with fGO exhibited a significantly better effect.

## 4. Conclusions

The use of graphene oxide or functionalized graphene oxide as an effective synergistic agent for the FRPU system improved the flame retardancy of EG/DMMP/RPUF composites. The thermogravimetric analysis results showed that there were increments in *T_ini_* and residue mass for the FRPU/GO and FRPU/fGO specimens compared with those of FRPU under both the air and nitrogen atmosphere. Furthermore, the TG kinetics results showed that the *E_a_* of FRPU/GO and FRPU/fGO were much higher than those of FRPU, and had values of 170 kJ·mol^−1^ and 191 kJ·mol^−1^, respectively. The FT-IR analysis of residues showed that the quantity of flammable and nonflammable products increased at high temperature. Therefore, it can be seen that the thermal stability of nanocomposites improved obviously. From the microstructure of the nanocomposites, it was found that GO and fGO enhanced the char layer and made it continuous and compact. Additionally, the structural strength of residues is significantly higher than that of FRPU system. The formation of a continuous and compact carbon shield effectively inhibits the release of heat and combustible organic volatiles. The flame retardancy of FRPU system containing GO and fGO obviously improved. 

## Figures and Tables

**Table 1:** Formulations containing different additive levels, limiting oxygen index (LOI) value, and UL-94 rating of the specimens.

Sample	Polyether Polyol (g)	Isocyanate (g)	EG (phr)	DMMP (phr)	GO (phr)	fGO (phr)	LOI (%)	UL-94 Rating
RPUF	50	50	–	–	–		19.0	No rating
FRPU	50	50	7.5	2.5	–		26.5	V-1
FRPU/GO	50	50	7.5	2.5	0.25		27.5	V-0
FRPU/fGO	50	50	7.5	2.5		0.25	28.1	V-0

**Table 2:** Typical thermogravimetric analysis (TG) parameters of flame retardant thermosets.

Sample	Air					Nitrogen		
	*T_ini_* (°C)	*T_max_* (°C)			Residue at 700 °C (%)	*T_ini_* (°C)	*T_max_* (°C)	Residue at 700 °C (%)
		*T*_1*max*_	*T*_2*max*_	*T*_3*max*_				
RPUF	281	134	317	541	0.5	276	349	15.9
FRPU	257	161	320	549	7.0	256	345	20.3
FRPU/GO	260	163	319	548	9.5	269	346	22.7
FRPU/fGO	269	164	321	551	12.5	278	347	24.4

**Table 3:** Activation energies (*E_a_*) of the RPUF specimens.

Sample	Heating Rate, Φ (°C·min^−1^)	*T_m_* (°C)	Activation Energy, *E_a_* (kJ·mol^−1^)
RPUF	5	326.6	130
	10	347.0	
	15	349.3	
	20	358.0	
FRPU	5	325.6	128
	10	338.2	
	15	346.1	
	20	357.7	
FRPU/GO	5	330.2	170
	10	338.0	
	15	348.2	
	20	353.3	
FRPU/fGO	5	330.3	191
	10	339.3	
	15	346.7	
	20	351.8	
